# Identification of genes associated with spontaneous regression of neuroblastoma

**DOI:** 10.1002/pdi3.10

**Published:** 2023-06-09

**Authors:** Yunlong Zhang, Yifei Du, Min Wang, Changchun Li, Zhenzhen Zhao, Liang Peng, Jianwu Zhou, Shan Wang

**Affiliations:** ^1^ Department of Pediatric Surgical Oncology Children's Hospital of Chongqing Medical University National Clinical Research Center for Child Health and Disorders Ministry of Education Key Laboratory of Child Development and Disorders Chongqing Key Laboratory of Pediatrics Chongqing China

**Keywords:** differentially expressed genes (DEGs), LASSO regression analysis, neuroblastoma (NB), spontaneous regression

## Abstract

The study of target genes for the spontaneous regression phenomenon of neuroblastoma (NB) is still unclear. Common differentially expressed genes (DEGs) were identified by differential expression analysis in both public databases for the stage 4 death group and stage 4S survival group. The DEGs were ranked by constructing protein–protein interaction (PPI) network as well as calculating betweenness centrality (BC) values, and the relationship with NB prognosis was determined by performing univariate analysis, multifactor analysis, and lasso regression analysis on the top 10 genes and possibly correlated with spontaneous regression of NB. We identified a total of 173 DEGs, including 143 upregulated genes and 30 downregulated genes. PPI network showed a rich interaction between DEGs. We ranked the DEGs by calculating BC values and showed the top 10 genes, followed by univariate and multifactorial analyses, which showed that *GRIA2*, *NTRK1*, *SCN9A*, *SLC18A2*, *CNR1*, *PIK3R1*, and *DGKB* were associated with good prognosis, and *CNR1* was the most closely associated with prognosis among them. By lasso regression analysis, we constructed a four‐gene risk score formula. We drew a nomogram to use in clinical work and two newly identified genes associated with good prognosis in NB: *CNR1* and *GIRA2*.

## Graphical abstract

By screening differential genes between the stage 4 death group and the stage 4 survival group of neuroblastoma in a public database, a group of genes that may be related to the spontaneous regression phenomenon of neuroblastoma was identified. A four‐gene risk scoring formula and nomogram have been established to assist clinical work.

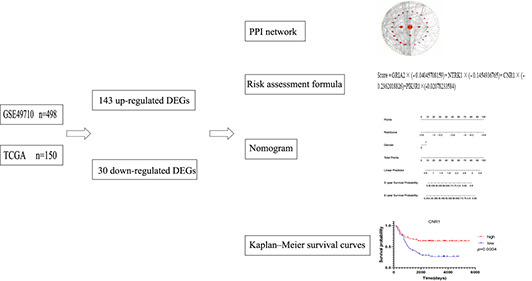

## 1 INTRODUCTION

Neuroblastoma (NB) is an early childhood developmental tumor that originates from the embryonic sympathetic‐adrenal spectrum of the neural crest. NB is the leading cause of death from childhood cancer in children aged from 1 to 5 years^1^ and its clinical presentation and course varies according to Tumor Biology. The unique features of this neuroendocrine tumor are the early age of onset, high frequency of metastatic disease at diagnosis, and tendency for spontaneous regression of the tumor in infancy.^2^ NB stage 4S is characterized by a high rate of spontaneous tumor regression and long‐term survival rates are estimated between 65% and 92%.^3^, ^4^, ^5^ Spontaneous regression refers to the reduction or disappearance of primary or metastatic diseases without treatment intervention. Indeed, previous large‐scale screening programs in Japan, Quebec, and Europe have documented the prevalence of spontaneous regression of NB.^6^, ^7^, ^8^, ^9^ Recently, a single‐cell landscape analysis of primary NB revealed the ability of neuroendocrine cells to differentiate into benign fibroblasts with highly expressed *CCL2* and *ZFP36*,^10^ which provides a new orientation for spontaneous regression of NB.

Presently, there is evidence to support several possible mechanisms of spontaneous NB degeneration, including (1) neurotrophin receptors, (2) immunological mechanisms, (3) telomerase and telomeres, and (4) epigenetic regulation and other mechanisms. Tropomyosin receptor kinase inhibitors, including GNF‐4256^11^ and CEP‐701,^12^ have shown initial success in initiating the process of apoptosis and degeneration of tumors. Therefore, more in‐depth study of the mechanism of spontaneous regression of NB will also help to explore new therapeutic directions. In this study, we review some of the mechanisms and identify a set of genes that may be associated with spontaneous regression of NB by screening public databases for differential expression analysis of stage 4 death cases and 4S survival cases. In addition, we construct a risk score formula for the four genes and create a nomogram to use in clinical work.

## 2 METHODS

GSE49710 was obtained from the Gene Expression Synthesis (GEO) database (http://www.ncbi.nlm.nih.gov/geo/). The Cancer Gene Atlas (TCGA) dataset was downloaded from the publicly available TCGA Data Portal at https://tcga‐data.nci.nih.gov/tcga/. Data were analyzed using the software SPSS, GraphPad Prism 8, Cytoscape, and R (version 4.1.3). The R packages “stringr,” “org.Hs.eg.db,”“enrichplot,” “pathview,” “Formula,” “ggplot2,” and “DOSE” were applied. Samples with both corrected *P* value < 0.05 and |log fold changes (FC)| > 1 were deemed to be the differentially expressed genes (DEGs). Venn diagram was built using online site (http://bioinformatics.psb.ugent.be/webtools/Venn/). STRING is an online platform to build protein–protein interaction (PPI) networks (https://cn.string‐db.org/). A receiver operating characteristic (ROC) curve analysis was performed to analyze the area under the ROC curve (AUC). A survival analysis was performed using the Kaplan–Meier method to compare survival differences. The results of univariate–multivariate analyses were expressed as hazard ratios (HR) and 95% confidence intervals (CI), where *p* value < 0.05 was considered statistically significant.

## 3 RESULTS

### 3.1 Identification of DEGs

**FIGURE 1:**
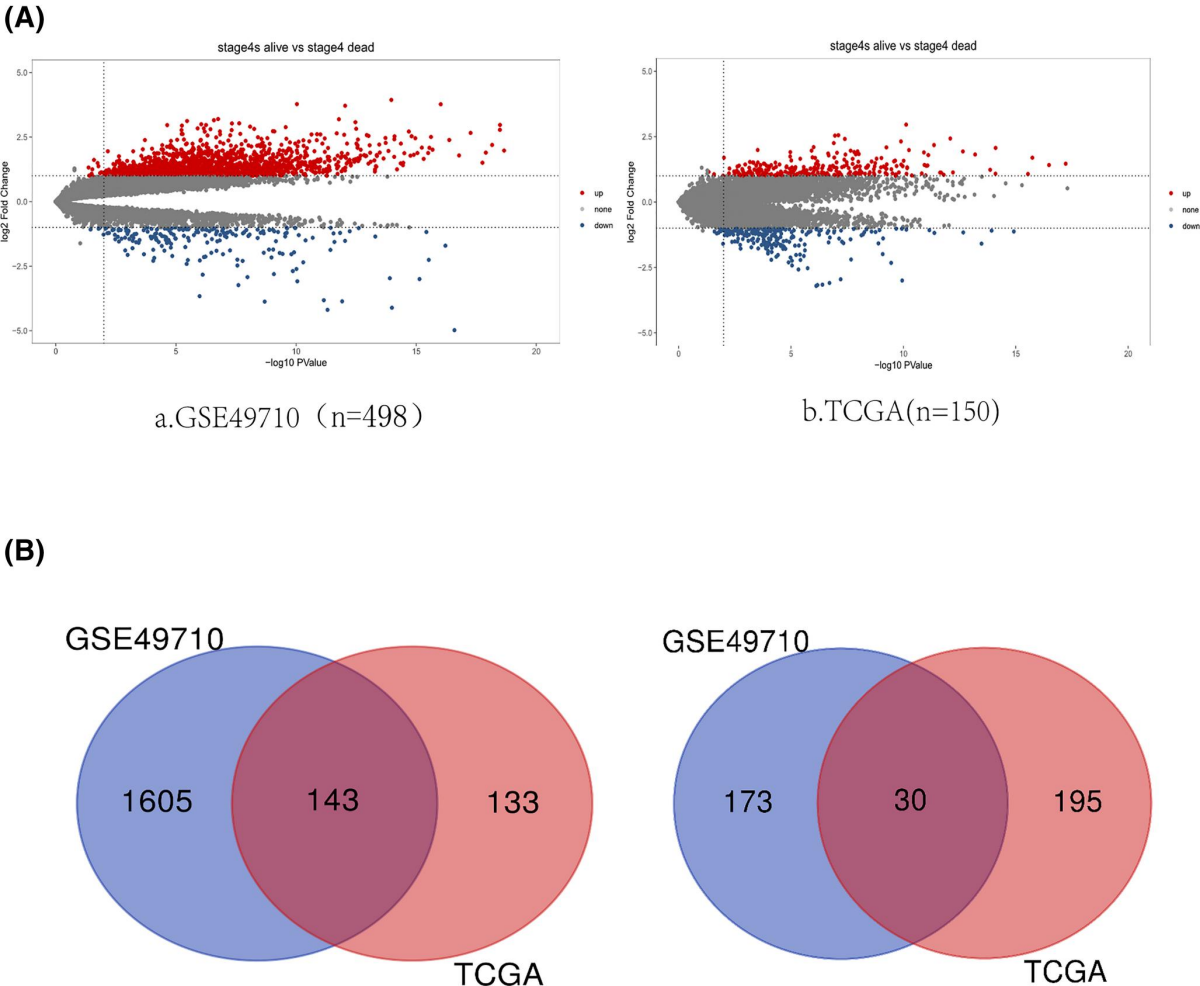
(A) Volcano plot distribution of DEGs between GSE49710 and TCGA (*n* = 150). Red dots in the volcano diagram represent common upregulated genes between groups and blue dots represent common downregulated genes between groups. (B) Venn diagram of 143 upregulated DEGs and 30 downregulated DEGs between GSE49710 and TCGA (*n* = 150). DEGs, differentially expressed genes; TCGA, The Cancer Genome Atlas.

To search for genes associated with spontaneous regression of NB, we considered patients with stage 4S who survived as a potential group that showed regression and patients with stage 4S who died as a group that could not show regression by comprehensive bioinformatics analysis in order to find core genes. One gene expression datasets (GSE49710) were obtained from the NCBI GEO database, the other one gene expression dataset was obtained from TCGA (*n* = 150). *p* value < 0.05, |log2FC| > 1, which were considered as the evaluation criteria for differential genes (Figure 1A). First, we used various packages in R, including limma package, to calculate the two datasets and selected common up‐ and downregulated genes using the Venn diagram, in which 173 DEGs (Table S1) were obtained, including 143 upregulated genes and 30 downregulated genes (Figure 1B).

### 3.2 Functional enrichment analysis of DEGs

**FIGURE 2:**
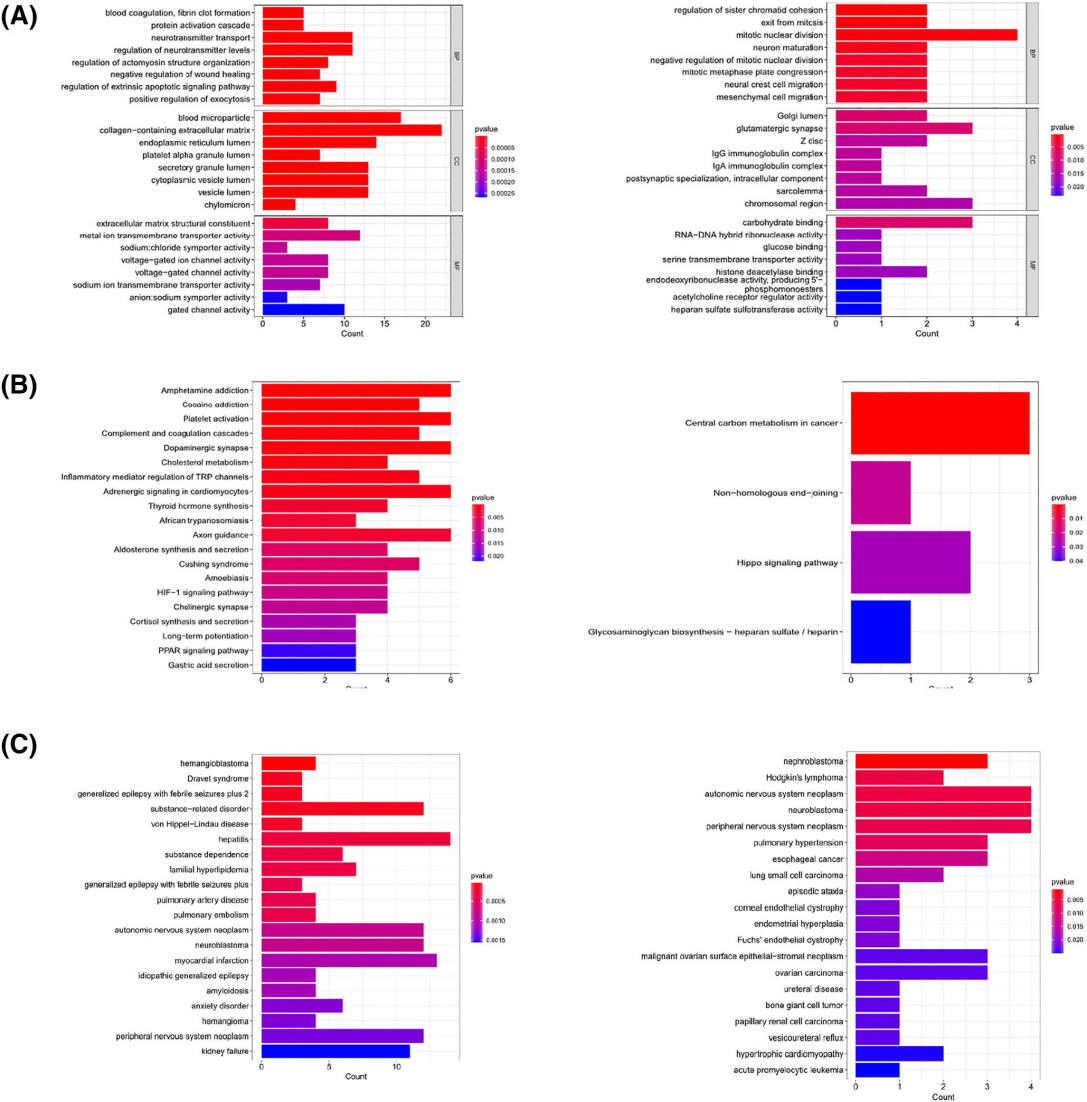
(A) GO enrichment analysis of upregulated genes (left) and downregulated genes (right). (B) KEGG enrichment analysis of upregulated genes (left) and downregulated genes (right). (C) DO enrichment analysis of upregulated (left) and downregulated genes (right). DO, Disease Ontology; GO, Gene Ontology; KEGG, Kyoto Encyclopedia of Genes and Genomes.

To further clarify the biological effects of DEGs and the pathways in which they are located, we conducted Gene Ontology (GO) and Kyoto Encyclopedia of Genes and Genomes (KEGG) analyses. The *P* value < 0.05 was considered significantly enriched. The GO functional enrichment analysis includes three main aspects: biological process, cellular component, and molecular function. Firstly, GO, KEGG, and Disease Ontology (DO) enrichment analyses were performed for the upregulated and downregulated genes, respectively. The results showed that for upregulated differential genes, the GO enrichment analysis mainly focused on neurotransmitter transport, regulation of neurotransmitter levels and collagen‐containing extracellular matrix (Figure 2A); for down‐regulated differential genes, GO enrichment analysis mainly focused on the mitochondrial nuclear division. For upregulated differential genes (Figure 2A), KEGG enrichment analysis focused on amphetamine addiction, platelet activation, and dopaminergic synapse, and also thyroid hormone synthesis and *HIF‐1* signaling pathway; for downregulated differential genes, KEGG enrichment analysis focused on central carbon metabolism in cancer (Figure 2B). DO enrichment analysis of upregulated differential genes mainly focused on hepatitis, substance‐related disorder, myocardial infarction, and NB, and DO enrichment analysis of downregulated differential genes mainly focused on NB, autonomic nervous system neoplasm, and peripheral nervous system neoplasm (Figure 2C).

### 3.3 Construction of PPI network and identification of core genes

**FIGURE 3:**
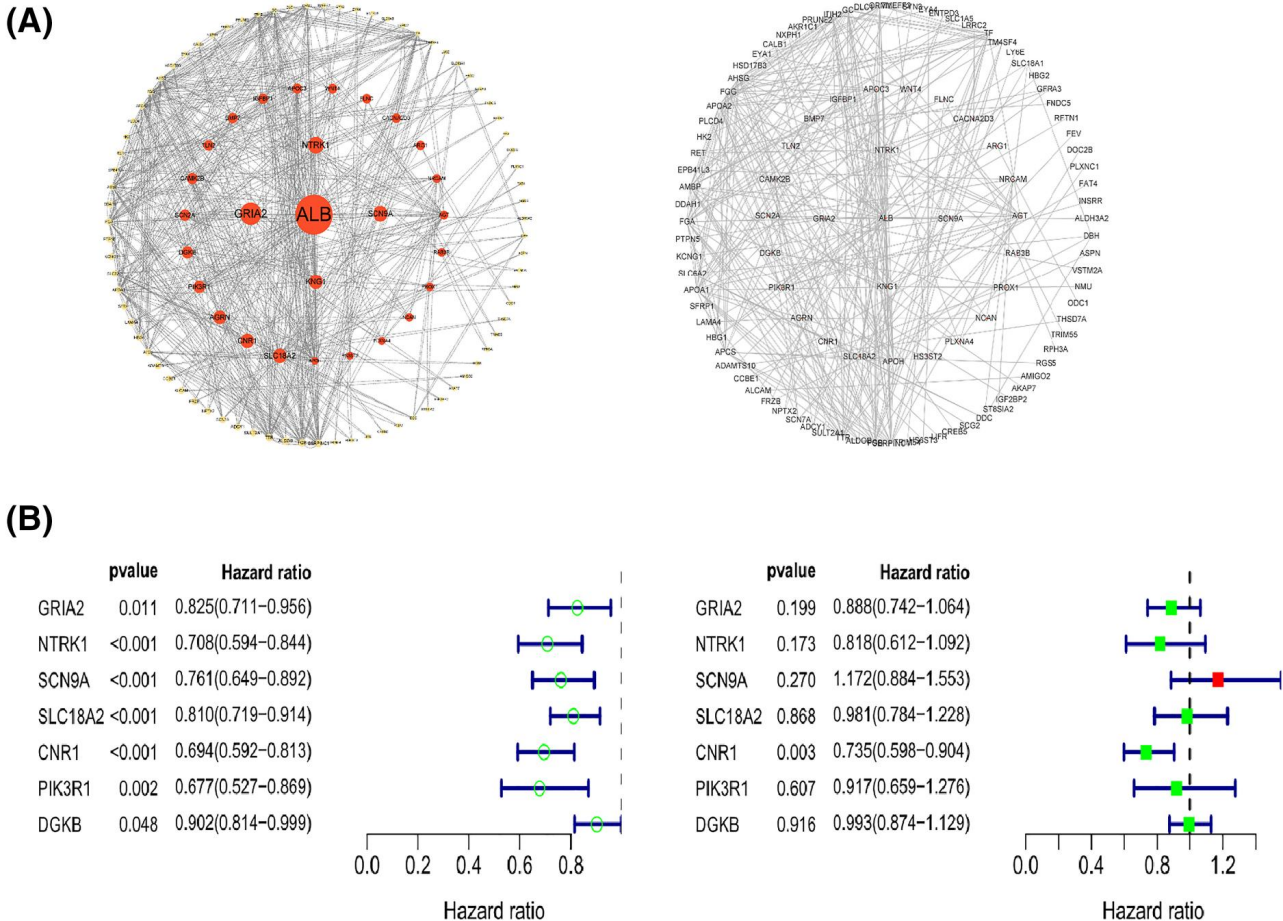
(A) PPI network of DEGs (left) larger circles represent larger BC values, (right) gene symbol of DEGs in PPI network. (B) Univariate Cox analysis and multivariate Cox analysis of TOP 10 genes in PPI network. BC, betweenness centrality; DEGs, differentially expressed genes; PPI, protein–protein interaction.

STRING is an online platform to build PPI networks based on DEGs to visualize protein–gene interactions. In this study, the minimum required interaction score was set to 0.4 in STRING. Based on the file obtained by inputting the previously obtained DEGs into STRING, we have successfully built the PPI network, then we used the CytoNCA algorithm in Cytoscape (version 3.9.1) to calculate the betweenness centrality (BC) of the DEGs. BC is widely used to identify key nodes in a network by exploring the ability of all nodes to act as intermediaries for information exchange.^13^ The size of a BC value can identify key nodes in the network. The larger the BC value, the stronger the interaction ability of the locus in the entire network, which means it will be in the core position; the BC values were normalized using SPSS software. By calculation, Table 1 shows the top 10 genes with BC values, which are all upregulated genes, namely *ALB*, *GRIA2*, *NTRK1*, *SCN9A*, *KNG1*, *SLC18A2*, *CNR1*, *AGRN*, *PIK3R1*, and *DGKB*. In the construction of PPI network in Cytoscape, we first remove the proteins that cannot form interactions, and then we build the network based on the ranking of BC value's size, the closer the inner layer of the circle, the larger the BC values (Figure 3A). The dashed line in the figure represents the protein interactions (Figure 3A). Therefore, we used these 10 genes as candidates for the next step of analysis.

**TABLE 1:** The BC values of top 10 DEGs.

	Gene symbol	BC values
1	ALB	7.47396
2	GRIA2	3.41823
3	NTRK1	2.11280
4	SCN9A	1.85694
5	KNG1	1.61761
6	SLC18A2	1.56296
7	CNR1	1.54557
8	AGRN	1.40894
9	PIK3R1	1.10320
10	DGKB	1.04685

Abbreviations: BC values, betweenness centrality values; DEGs, differentially expressed genes.

Next, to verify whether the selected gene set was associated with the prognosis of children with NB and to identify the core gene, we performed univariate analysis as well as multivariate analysis of candidate genes in the Therapeutically Applicable Research to Generate Effective Treatments database (*n* = 246) in R. *p* value < 0.05 was considered statistically significant. The univariate Cox analysis showed that *GRIA2*, *NTRK1*, *SCN9A*, *SLC18A2*, *CNR1*, *PIK3R1*, and *DGKB* were all associated with good prognosis of NB and HR was <1, while *ALB*, *KNG1*, and *AGRN* were not statistically significant and therefore not shown in the forest plot (Figure 3B). Multivariate Cox analysis showed that *CNR1* (*p* = 0.003, HR = 0.735; 95% CI = 0.598−0.904) was most strongly associated with prognosis among the seven previously described genes (Figure 3B).

**FIGURE 4:**
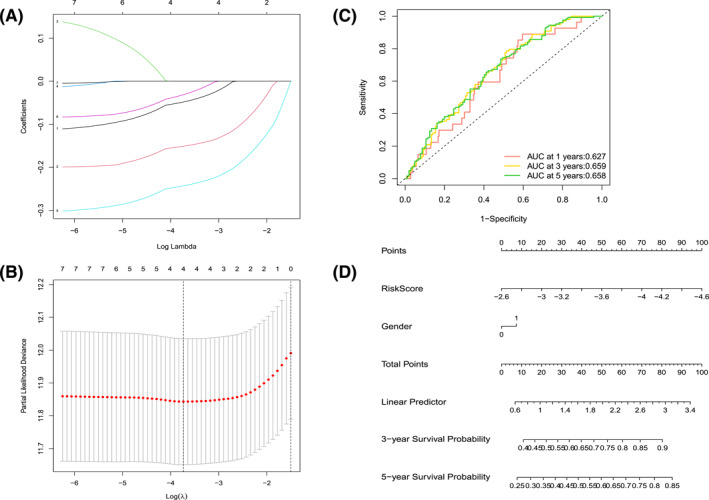
(A) Plot of LASSO regression coefficients. (B) Cross validation plot for the penalty term. (C) ROC curves. (D) Nomogram based on risk score calculated by lasso regression analysis. ROC, receiver operating characteristic.

To further filter the core genes as well as construct a predictive prognostic model, we performed lasso regression analysis on 7 candidate genes from the univariate Cox analysis. Since the number of genes corresponding to the lambda.1se value is zero, we choose the four genes corresponding to the lambda.min value as the core genes and construct the risk score calculation formula based on them (Figure 4B). The risk assessment formula is as follows.

Score=GRIA2×(−0.04045708159)+NTRK1×(−0.1454936765)+CNR1×(−0.2362018826)+PIK3R1×(−0.02078233584)

**FIGURE 5:**
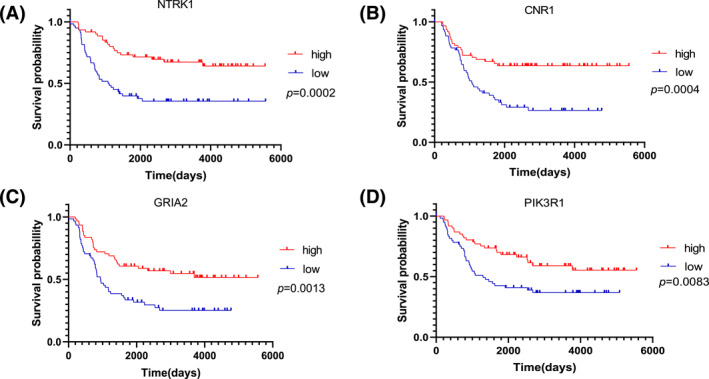
Kaplan–Meier survival curves for overall survival in a total of 246 patients with NB according to (A) NTRK1. (B) CNR1. (C) GRIA2. (D) PIK3R1.

We plotted the Kaplan–Meier survival curves of the four genes using GraphPad Prism 8. We considered the top 25% of gene expression as the high expression group and the bottom 25% of gene expression as the low expression group, and the results showed that high expression of all four genes was associated with a good prognosis of NB (Figure 5). Next, we performed ROC curve analysis to calculate the AUC. The AUC values of 1‐year survival, 3‐year survival, and 5‐year survival were 0.627, 0.659, and 0.658, respectively (Figure 4C). Then, we constructed the nomogram based on the risk score (Figure 4D). The nomogram showed the results of the calculations based on the previous four‐gene risk score formula; the smaller the risk score, the higher the predicted survival rate of the patient. For gender in the nomogram, 1 refers to male.

## 4 DISCUSSION

NB is the most common malignant extracranial solid tumor in childhood. NB demonstrates a remarkable ability to spontaneously regress and based on existing experience with large‐scale screening, there is still a proportion of degenerated tumors that have not been observed. In addition to stage 4S, spontaneous regression has been observed in cases of stage 3.^14^ Since the first report of spontaneous regression of tumors in 1956,^15^ the study of spontaneous regression of NB has entered the multi‐omics era. Nondependent programmed cell death of the cystathionase cascade may be associated with spontaneous regression of NB.^16^ A previous study identified a set of survival‐related long noncoding RNAs that may be associated with spontaneous regression of NB.^17^ In 2016, the first study of the DNA methylome of stage 4S revealed the unique methylation pattern of stage 4S^18^; in 2022, high expression of E2 transcription factor 3 (*E2F3*) was associated with poorer survival in 134 stage 4S patients,^19^ and these will contribute to the study of spontaneous regression of NB.

To further investigate the mechanism of spontaneous regression of NB, we used public databases and applied bioinformatics to analyze the potential core genes. The results showed that we screened a total of 173 DEGs, including 143 upregulated genes and 30 downregulated genes. GO and KEGG enrichment analyses of upregulated DEGs showed that DEGs were mainly associated with neurotransmitter and collagen‐containing extracellular matrix and also included *HIF‐1* signaling pathway and *PPAR* signaling pathway. PPI network shows rich interaction between DEGs; we ranked the DEGs by the calculation of BC values and used the top 10 ranked genes as candidate genes. Next, we used lasso regression analysis to establish a four‐gene (*GRIA2*, *NTRK1*, *CNR1*, and *PIK3R1*) based risk score calculation formula and construct a nomogram. All four genes are associated with a good prognosis.

*GRIA2*, glutamate ionotropic receptor *AMPA* type subunit 2, is currently found to have reduced transcript editing in patients with glioblastoma^20^ and may be associated with perineural invasion of endometrial cancer (EC).^21^ The current study found that CNR1 (cannabinoid receptor 1) expression was elevated in human papillomavirus (HPV)‐positive head and neck squamous cell carcinoma (HNSCC) compared to HPV‐negative HNSCC,^22^ and *CNR1* also had a negative correlation with overall survival in EC.^23^ A previous study developed a three‐gene predictive model for NB prognosis including *PIK3R1* (phosphoinositide‐3‐kinase regulatory subunit 1).^24^ *TrkA* (encoded by *NTRK1*) is currently thought to be involved in the pathogenesis of NB and may be closely related to the spontaneous regression of NB. At present, the research of *GRIA2* and *CNR1* in NB has not been retrieved.

In a prospective trial, spontaneous regression occurred in 44 out of 93 patients who did not undergo tumor resection, a rate of 47%, even though some patients experienced transient tumor growth.^25^ We therefore have a theoretical basis for treating patients surviving stage 4S as a potential population experiencing spontaneous regression. We identified seven potential target genes, including *NTRK1*, by screening with BC values and univariate regression analysis. A four‐gene risk assessment formula was created based on this, in which the specific roles of *GRIA2* and *CNR1* in NB are not yet known, but can be clearly associated with a good prognosis.

As a convenient and intuitive prediction tool, nomogram has been widely used in the study of NB, therefore, we transformed the more complex risk score calculation formula into a nomograph to use in clinical work.

However, the present study still has some drawbacks. First, we considered patients who died at stage 4 as not experiencing spontaneous regression, which obviously leads to a reduced sample size because patients who died at the remaining stages also did not experience spontaneous regression phenomenon; therefore, further studies are needed to expand the scope of the study. In addition, if we could obtain tumor samples that experienced spontaneous regression and perform experiments to verify gene expression, this would certainly increase the credibility of the study.

In conclusion, we identified a set of genes that may be associated with spontaneous regression of NB by screening public databases and constructed a four‐gene risk assessment formula based on this, with all four genes associated with a good prognosis of NB. Nomogram serves as a predictive tool to conveniently predict the survival of NB patients and helps to individualize treatment.

## AUTHOR CONTRIBUTIONS

Shan Wang was responsible for the overall idea of the project and guiding the direction of the research, as well as revising the manuscript; Yunlong Zhang was responsible for the overall data analysis and completed the manuscript; Min Wang and Yifei Du completed some of the data analysis; Changchun Li, Zhenzhen Zhao, Liang Peng, and Jianwu Zhou contributed to part of the guidance of this study.

## CONFLICT OF INTEREST STATEMENT

The authors declare no conflicts of interest.

## ETHICS STATEMENT

This article uses public databases in which the patients involved have received ethical approval. Users are free to download relevant data for research and publication. Our research is based on open source data and is therefore free of ethical issues and other conflicts of interest.

## Supporting information

Table S1

## Data Availability

The data that support the findings of this study are available from the corresponding author upon reasonable request.
